# Safety Profile, In Vitro Anti-Inflammatory Activity, and In Vivo Antiulcerogenic Potential of Root Barks from *Annona senegalensis* Pers. (Annonaceae)

**DOI:** 10.1155/2021/4441375

**Published:** 2021-09-06

**Authors:** Kiessoun Konaté, Abdoudramane Sanou, Raïssa R. R. Aworet-Samseny, Fatiha Benkhalti, Oksana Sytar, Marian Brestic, Alain Souza, Mamoudou Hama Dicko

**Affiliations:** ^1^Laboratory Biochemistry, Biotechnology, Food Technology and Nutrition (LABIOTAN), Department of Biochemistry and Microbiology, University Joseph KI-ZERBO, 03 B. P. 7021, Ouagadougou, Burkina Faso; ^2^Applied Sciences and Technologies Training and Research Unit, Department of Biochemistry and Microbiology, University of Dedougou, B. P. 176, Dedougou, Burkina Faso; ^3^Institut Pharmacopoeia and Traditional Medicine, National Center for Scientific and Technological Research, P.O. Box: 1156, Libreville, Gabon; ^4^Laboratory of Bio-Organic and Macromolecular Chemistry Department of Biology, Faculty of Sciences and Techniques, B. P. 549, Marrakech, Morocco; ^5^Department of Plant Physiology, Slovak Agriculture University in Nitra, A. Hlinku 2 94976, Nitra, Slovakia; ^6^Laboratory of Animal Physiology, Electrophysiology and Pharmacology, Faculty of Sciences, University of Science and Technology of Masuku, Franceville-Gabon, Gabon

## Abstract

*Annona senegalensis* (Annonaceae) is a tropical shrub widely distributed in Burkina Faso. This plant is traditionally used as a medicine against many pathologies including typhoid fever, gastrointestinal disorders, ulcers, and inflammatory and infectious diseases. The present study was conducted to evaluate the anti-inflammatory and antiulcer properties of *Annona senegalensis* root bark extracts. Therefore, toxicity tests were first performed, followed by other biological tests. For this purpose, we first undertook to evaluate the toxicity tests before considering the other biological tests in a second step. The results showed that the extracted fractions had a significant effect for the different methods used (protein denaturation inhibition activity, hyaluronidase inhibition activity, and xanthine oxidase inhibition activity). However, of the extracted fractions used, the ethyl acetate fraction was the most anti-inflammatory fraction. The antiulcer activity was evaluated using the best bioactive fraction. The antiulcer effect of the ethyl acetate fraction may be due to both the reduction of gastric acid secretion and gastric cytoprotection. The results of this study also showed that the bioactive fraction reduced ethanol-induced ulceration and pyloric ligation in a dose-dependent manner, and at the highest dose (200 mg/kg), the effect was similar to that of the reference drug. In summary, the ethyl acetate fraction was found to have the best anti-inflammatory and antiulcerogenic activities. The ethyl acetate fraction at a dose of 200 mg/kg also showed a rather interesting level of cytoprotection. The anti-inflammatory and antiulcer activities could be due to the different secondary metabolites contained in the fractions extracted from *Annona senegalensis*, notably flavonoids, triterpenoids, steroids, saponins, and tannins. As the mechanisms of action are still little or not understood, we will consider in the future identifying the phytoconstituents and the mechanisms of action involved in the results.

## 1. Introduction

Traditional herbal medicine has always been known to be used by humans [1]. The beneficial alternative use of traditional herbal medicine is the result of the failure of modern medicine to treat certain diseases accurately [2]. In many countries, herbal medicines are useful for certain conditions where modern medicine has been ineffective [3]. Ulcers are considered a serious condition among inflammatory and metabolic diseases. Nonsteroidal anti-inflammatory drugs (NSAIDs) are most commonly used in inflammatory diseases. However, these synthetic molecules are not adequate to treat all forms of inflammatory diseases. Indeed, these drugs can cause complications ranging from bleeding to ulcers [4]. Nowadays, the use of phytopharmaceuticals has become popular due to their analgesic and anti-inflammatory effects [5].

Chronic and recurrent gastric ulcers are a common gastrointestinal disorder. Some studies have shown that this disease affects at least 10% of the world's population [6, 7]. The causes of gastric ulcers are diverse, and the best known are related to successive stresses due to active consumption of tobacco, alcohol, caffeine, certain nonsteroidal anti-inflammatory drugs, and bacterial infections such as *Helicobacter pylori* infection [8]. Despite the presence of a large number of synthetic molecules in the treatment of various pathologies including gastric ulcers, traditional medicine also occupies a prominent place in the world for the treatment of gastric ulcers [9]. This can be explained by the persistence of side effects, drug interactions, microbial resistance, and the exorbitant costs of synthetic drugs [10]. Therefore, natural plant compounds and extracts with specific pharmacological properties and proven efficacy can be recommended as alternatives to synthetic molecules [11, 12].

A medicinal plant from Burkina Faso, *Annona senegalensis* is a widely distributed shrub in West Africa, and various parts of this shrub are used in the treatment of several conditions such as fever, intestinal disorders, stomachache, gonorrhoea, syphilis, rheumatism, gastric ulcers, and central disorders according to some ethnobotanical surveys [13]. Previous pharmacological studies have shown that this plant has antiepileptic effects [14]. It should be noted that any bioactive compound can cause short- or long-term toxic effects. Thus, it seems necessary to always carry out a toxicological study of each new substance or compound in order to prevent the risks that it could cause. Until now, no long-term toxicological studies and no research on the in vivo biological activity of the root bark have been carried out on the plant *Annona senegalensis*. Therefore, the aim of this research work was to evaluate the in vitro anti-inflammatory activity and the in vivo antiulcer activity of fractions and extracts of *Annona senegalensis.*

## 2. Materials and Methods

### 2.1. Study Area

The present study was conducted in the Boucle du Mouhoun region in Kari, 22 km from Dedougou. Dedougou is located about 230 km from Ouagadougou, the capital of Burkina Faso. The Boucle du Mouhoun region is located in the northwest of the country with Dedougou as its capital. This region covers 12% of the national territory with 34.497 km^2^ and 1494043 inhabitants representing 11.23% of the total population of the country according to the regional profile study [15].

### 2.2. Plant Material

Plant material including root barks of our study plant *Annona senegalensis* Pers. (Annonaceae) was collected in January 2015 in Kari, a village in the commune of Dedougou, in January 2015 (Burkina Faso). Dedougou is located about 230 km from Ouagadougou, the capital of Burkina Faso. For identification and authentication, we used the services of Professor Millogo Rasolodimby, botanist at the Laboratory of Biology and Ecology of the University of Ouagadougou. A reference specimen bearing the number 836/01/2015/KK was deposited in the herbarium of the Life and Earth Sciences Unit, University of Ouagadougou.

### 2.3. Extractions

About 50 mg of plant material was pulverised with 400 ml of acetone and 100 ml of distilled water, and the mixture was subjected to mechanical agitation (SM 25 shaker, Edmund BÜHLER, Germany) for 24 hours at 37 °C. After this agitation, the acetone was evaporated in a rotavapor (BÜCHI, Rotavopor R-200, Switzerland) at 40 °C. The aqueous extracts were then subjected to liquid-liquid fractionation of increasing polarity with n-hexane, dichloromethane, ethyl acetate, and n-butanol. Each fraction was concentrated to obtain fractionated extracts (n-hexane fraction (n-HF), dichloromethane fraction (DCMF), ethyl acetate fraction (EAF), and n-butanol fraction (n-BF)) after freeze-drying using the Telstar Cryodos 50 freeze-dryer [16], and then, the extraction yields were calculated by the following formula:

(1) $$\begin{array}{c} R=\left(\frac{A}{B}\right)\times 100, \end{array}$$

where *R* = extraction yields; *A* = mass of the extracted residue; *B* = mass of the plant powder.

### 2.4. Animals

20–30 g mice (Swiss NMRI) and 160–165 g albino rats (Wistar) of both sexes were used for the study. The animals were housed and fed under acceptable and recommended experimental conditions. Prior to the actual experiment, the animals were deprived of food and water for 15 hours and weighed. All experimental procedures were performed in accordance with the National Institute for Health (NIH) Guide to Animal Care [17]. Specifically, animal experiments were performed according to protocols implemented by the Institutional Ethical Review Board of the Health Sciences Research Institute, in accordance with the Guidelines for the Care of Laboratory Animals and the Ethical Guidelines for the Study of Experimental Pain in Conscious Animals [18].

### 2.5. Toxicological Studies

#### 2.5.1. Acute Toxicity Assessment

Male and female mice were divided into seven groups of four animals per group. Group 1 served as a control, and the other groups served as test groups. First, the animals were allowed to comply for a few minutes of adaptation before oral administration of the extract or DMSO. The control group received distilled water in 10% DMSO, and the test groups received increasing doses of the hydroacetone extracts of 100 mg/kg; 600 mg/kg; 1000 mg/kg; 2000 mg/kg; 3000 mg/kg; and 5000 mg/kg, respectively. For this experiment, the route of administration was chosen to be intraperitoneal. After administration of the extract at different doses, the general behaviour of the animals was observed for a period of 120 minutes. Then, morbidity and mortality were observed once a day for 14 days. The number of survivors after 14 days was counted and recorded. The toxicological effect was assessed on the basis of the mortality rate during the 14 days and was expressed as the median lethal dose value 50 or LD_50_, estimated by the log-probit mortality rate regression line [19].

#### 2.5.2. Assessment of Subacute Toxicity

Adult Wistar rats of both sexes were used for this purpose. The animals were divided into 4 groups of 4 animals of both sexes, two males and two females. The first group served as a control, and the animals received distilled water containing 10% DMSO. The other groups were tested and received daily and orally (gavage) for 28 days 100, 200, and 300 mg/kg of extract dissolved in 10% DMSO, respectively. For daily monitoring, the body weights of the animals were recorded weekly, and the animals were observed daily for any signs of abnormality throughout the study. On the 28^th^ day, the animals were deprived of food and water for 15 hours and anaesthetised under chloroform vapour, and blood samples were collected by cardiac puncture into EDTA-containing vials for biochemical and haematological analysis, respectively. The organs (liver, kidney, heart, and lungs) were removed and weighed [20].

*(1) Assessment of Relative Organ Weights*. The vital organs of the animals, namely, the heart, lungs, liver, and kidneys, were excised, deblooded, weighed, and examined macroscopically, and their relative weight and the percentage of the assessed relative organ weight were recorded (*P*%) and calculated as follows:

(2) $$\begin{array}{c} \left(P\%\right)=\left(\frac{A}{B}\right)\times 100, \end{array}$$

where *A* = weight of the animal organ and *B* = animal body weight.

*(2) Evaluation of Biochemical Parameters*. The biochemical parameters were determined from the serum obtained after centrifugation of blood collected from the animals in ordinary tubes. After collecting the blood, we then stored it for at least 3 hours to allow complete coagulation. The clotted blood samples were then subjected to a series of centrifugations at 3500 rpm for 10 minutes. The serum obtained was then used to determine biochemical parameters such as aspartate aminotransferase (AST), alanine aminotransferase (ALT), alkaline phosphatase (ALP), total protein, albumin, and direct and indirect total bilirubin. Kidney electrolytes, creatinine, and uric acid were determined. All these parameters were determined using standard kits (Randox Ltd., UK) following the manufacturer's instructions.

*(3) Assessment of Haematological Parameters*. An automated haematological analyser (Sysmex-XT-1800, Kobe, Japan) was used to assess haematological parameters such as total red blood cells (TRBCs), platelet count leukocytes (WBC), neutrophils, basophils, eosinophils, lymphocytes, monocytes, mean corpuscular volume (MCV), mean corpuscular haemoglobin (HMC), and mean corpuscular haemoglobin concentration (MCHC).

### 2.6. In Vitro Anti-Inflammatory Activity of Fraction Extracts

#### 2.6.1. Assessment of Inhibition of Protein Denaturation Activity

A spectrometric method with modifications [21] was used. For the reaction mixture, fraction extracts were used at the final concentration of 100 *µ*g/mL and 1% BSA (aqueous solution). 1 N HCl was used to adjust the pH of the mixture used. The different samples were kept at 37°C for 20 minutes, then at 57°C for 20 minutes, and cooled. The turbidity of the samples was also measured at 600 nm. Our experiment was repeated in triplicate. Inhibition as a percentage of protein denaturation and noted (*I*%) was calculated as follows:

(3) $$\begin{array}{c} I\%=\left(1-\frac{\text{AE}}{\text{AC}}\right)\times 100, \end{array}$$

where AC = absorbance of the control and AE = sample absorption. We had used aspirin as a reference substance.

#### 2.6.2. Assessment of Hyaluronidase Inhibitory Activity

A spectrometric method with modifications [22] was used. Fraction extracts were used at the final concentration of 100 *µ*g/mL and incubated with the hyaluronidase enzyme solution (10 *µ*L) at 37°C for 10 minutes, followed by the addition of calcium chloride (12.5 mM, 20 L) and reincubation at 37°C for 10 minutes. Next, sodium hyaluronate (50 *µ*L) was added to the final reaction mixture and incubated at 37°C for 40 minutes, followed by the addition of sodium hydroxide (0.9 M, 10 *µ*L) and sodium borate (0.2 M, 20 *µ*L) and, finally, incubation at 100°C for 3 min. p-Dimethylaminobenldehyde (PDMAB) (50 *µ*L, 67 mM) was added to the reaction mixture and incubated at 37°C for 10 minutes. Absorbance was measured at 585 nm. The percentage of enzymatic inhibition was noted (*I*%) expressed in the following formula:

(4) $$\begin{array}{c} I\%=\left(1-\frac{B}{A}\right)\times 100, \end{array}$$

where *A* = absorbance of the control and *B* = absorbance of the sample.

#### 2.6.3. Assessment of Xanthine Oxidase Inhibitory Activity

For this evaluation, the kinetic method [23] with slight modifications was used to evaluate the xanthine oxidase inhibitory activity of the fraction extracts. We used fraction extracts at the final concentration of 100 *µ*g/mL. Sodium phosphate buffer (150 *µ*L, 50 mM, pH 7.4), fraction extracts (10 *µ*L), and xanthine oxidase solution (10 *µ*L) were incubated at 25°C for 10 minutes. The reaction was continued with the addition of xanthine solution (0.1 mM). Absorption was monitored with the change in absorption at 295 nm for 15 min at 25°C. The percentage inhibition noted (*I*%) of xanthine oxidase was expressed using the following formula:

(5) $$\begin{array}{c} I\%=\left(1-\frac{C}{D}\right)\times 100, \end{array}$$

where *D* is the activity of the enzyme without extracts and *C* is the activity of the enzyme with extracts. Allopurinol was used as the reference substance.

### 2.7. In Vivo Antiulcerogenic Potential of the Bioactive Fraction

After the antioxidant activities, it was revealed that the ethyl acetate fraction (EAF) was the best fraction compared to the others (bioactive fractions). Therefore, our different activities should be evaluated with this fraction.

#### 2.7.1. Assessment of Antisecretory Activity by Pyloric Ligation-Induced Ulcers

Antiulcer activity was applied using the antisecretory method by pyloric ligation of the pyloric end of the stomach used according to the work in [24] with slight modifications. Four groups of adult Wistar albino rats of both sexes were used, and each group had six rats. Group 1 served as a normal control (vehicle) and received 0.5% carboxymethyl cellulose (CMC) and group 2 served as a standard (omeprazole at the dose of 20 mg/kg), while animals in groups 3 and 4 received doses of 100 and 200 mg/kg, respectively, of the ethyl acetate fraction (EAF) of root barks of *Annona senegalensis* Pers. (Annonaceae) daily for 3 days.

The animals were fasted overnight prior to the start of the experiment and given water ad libitum. Pyloric ligation was applied by precisely ligating the pyloric end of the stomach of the rats on day 3 under phenobarbital anaesthesia at a dose of 35 mg/kg b.w., after 30 minutes of fraction extract or omeprazole treatment. The animals were allowed to recover and stabilise in an individual cage and were deprived of water during the postoperative method after 4 hours of surgery. Rats were sacrificed with an overdose of ether; the stomachs were removed, and gastric juices were collected for the study of gastric secretion and stomach ulcer scoring as described in [25]. Gastric juices were collected and centrifuged. Their volume and pH were recorded and subjected to biochemical estimates such as free acidity and total acidity. The ulcer score was divided by a factor of 10 to obtain the ulcer index and scored (UI). The percentage of ulcers classified as “protective” (P%) was evaluated as follows:

(6) $$\begin{array}{c} \left(P\%\right)=\left(1-\frac{{\text{IU}}_{\text{S}}}{{\text{UI}}_{\text{C}}}\right)\times 100, \end{array}$$

where UI_C_ = ulceration index of control means and IU_S_ = ulceration index of test means.

*(1) Gastric Fluid*. The different biochemical parameters such as secretions, gastric volume, pH, and free and total acidity were evaluated.

(1) Gastric volume:
The gastric volume was measured after centrifugation of the gastric fluid; it was left to stand, decanted and poured into the 0.01 ml graduated cylinder.
(2) Determination of pH:
The pH was determined using a pH meter (Cyber Scan, India).
(3) Assessment of free and total acidity:
Free acidity and total acidity were assessed according to the work of Suzuki et al. [26]. For this, 1 ml of gastric juice was pipetted into a 100 ml conical flask. Two to three drops of Topfer's reagent were added and titrated with 0.01 N sodium hydroxide (NaOH), previously standardised with 0.01 N oxalic acid. The mixture was then observed until all traces of red colour had disappeared and the colour of the solution was yellowish. The volume of alkali was noted. This volume, therefore, corresponds to the free acid. Two or three drops of phenolphthalein solution were also added, and the titration was continued until a definite red tinge reappeared. Again, the total volume of alkali added was noted. This volume corresponds to the total acidity. Finally, the acidity, noted A, was calculated using the following formula:
  (7) $$\begin{array}{c} A=\frac{\left(V\times N\times 100\;\text{meq}/\text{lt}/100\text{ g}\right)}{\text{0.}1}, \end{array}$$
where *V*  = volume of NaOH and *N*  = actual normality of NaOH.

#### 2.7.2. Gastric Ulcer By Ethanol-Induced Ulcer Assessment

The antiulcer activity using the cytoprotective model was performed according to the work in [27] with some modifications. After 12 hours of fasting, albino Wistar rats of both sexes were used and divided into five groups of six animals each. Group 1 served as a normal control (vehicle) and received 0.5% carboxyl methylcellulose (CMC), group 2 served as an ethanolic control, and group 3 was treated with a standard (omeprazole at 20 mg/kg). Test groups 4 and 5 received 100 and 200 mg/kg ethyl acetate fraction (EAF) of *Annona senegalensis* Pers. (Annonaceae), respectively. We used the oral route for administration. After 1 hour of oral treatment, each rat received absolute ethanol (5 mL/kg) orally and was kept for another 1 hour. Then, the animals were subjected to an ether overdose, and their stomachs were immediately excised. Each stomach was opened along the greatest curvature and washed with distilled water. The gastric mucosa was carefully examined with a magnifying glass to identify ulcers, and ulcer scoring was performed according to the work in [28]. The average ulcer score for each animal was expressed as the ulcer index. The percentage of protection against ulcers was assessed using the following formula:

(8) $$\begin{array}{c} \left(P\%\right)=\left(1-\frac{{\text{IU}}_{\text{S}}}{{\text{UI}}_{\text{C}}}\right)\times 100, \end{array}$$

where UI_C_ = average ulcer index of the control and IU_S_ = average ulcer index of the test.

### 2.8. Statistical Analysis

Quantitative data were subjected to descriptive statistics and expressed as mean ± standard error of the mean (SEM) with (*n* = 3). In vivo data were expressed as mean ± standard deviation (SD), and values are mean ± SEM (*n* = 6). All results were analysed by one-way ANOVA followed by Dunnett's *t*-test using Prism 4 software. The significance level was considered at *P* ≤ 0.05.

## 3. Results

### 3.1. Extraction Yields

The extraction yields were evaluated from hydroacetonic extract (HAE) and the different extract fractions ((*n*-hexane fraction (*n*-HF), dichloromethane fraction (DCMF), ethyl acetate fraction (EAF), and n-butanol fraction (*n*-BF)), respectively. The best yield was obtained with the hydroacetone extract, followed by the dichloromethane fraction, then the ethyl acetate fraction, then the butanol fraction, and finally, the hexane fraction with, respectively, 19.12% (HAE); 14.51% (DCMF); 13.88% (EAF); 12.06% (BF); and 8.10% (HF). To perform all the biological tests, the extractions were repeated several times.

### 3.2. Acute Toxicity Test

For acute toxicity, hydroacetonic extract of *Annona senegalensis* Pers. root barks produced no lethality or visible signs of toxicity in animals up to an oral dose of 3400 mg/kg body weight 24 hours after treatment. Closer monitoring for seven days still did not result in mortality or visible signs of toxicity. Therefore, the LD_50_ value was greater than 3400 mg/kg body weight.

### 3.3. Subchronic Toxicity Study in Rats

#### 3.3.1. Body Weight

**Figure 1:**
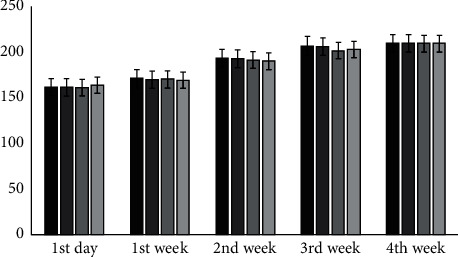
Effects of hydroacetonic extract on the body weight of the treated rats. No statistical difference (*P* > 0.05) was found compared with the control group.

No significant difference in body weight gain between the control and test groups was observed during the first days of treatment (*P* > 0.05). However, during four weeks, a significant decrease in body weight was observed between the test and control groups (*P* < 0.01). The results are summarised in Figure 1.

#### 3.3.2. Effects of Hydroacetonic Extract of Root Barks from *Annona senegalensis* Pers. on Relative Organ Weight of Rats

**Figure 2:**
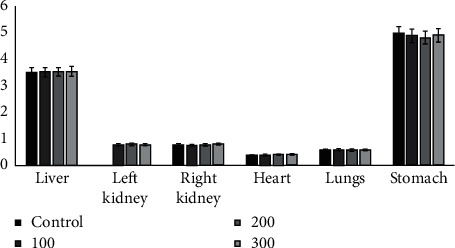
Effect of hydroacetonic extract on albino rats' relative weight of vital organs weight. No statistical difference (*P* > 0.05) was found compared with the control group.

The relative weights of the vital organs of the rats treated with the extract did not vary significantly compared to the control group. No statistical difference (*P* > 0.05) was found compared to the control group (Figure 2).

#### 3.3.3. Effects on Haematological Indices

**Figure 3:**
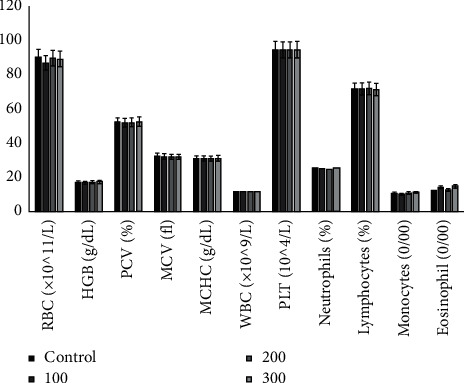
Effects of hydroacetonic extract of root barks from *Annona senegalensis* Pers. on rat haematological indices. RBC, red blood cells; HGB, haemoglobin; PCV, packed cell volume; MCV, mean corpuscular volume; MCHC, mean corpuscular haemoglobin concentration; WBC, white blood cells; and PLT, platelets. No statistical difference (*P* *>* 0.05) was found compared with the control group.

Figure 3 describes the effects of hydroacetonic extract of *Annona senegalensis* Pers. root bark on haematological indices. No significant differences were noted between the haematological parameters of rats exposed to all doses of the aqueous acetone extract of *Annona senegalensis* Pers. root barks compared to the control. There was no statistical difference (*P* > 0.05) compared to the control group.

#### 3.3.4. Effects on Biochemical Parameters

**Figure 4:**
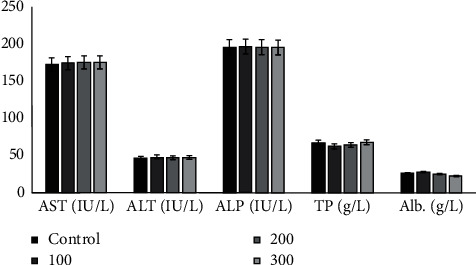
Effects of hydroacetonic extract of root barks from *Annona senegalensis* Pers. on rat liver function parameters. AST, aspartate aminotransferase, ALT, alanine aminotransferase; ALP, alkaline phosphatase; TP, total protein; and Alb, albumin. No statistical difference (*P* *>* 0.05) was found compared with the control group.

*(1) Liver Enzymes and Proteins*. The effects of hydroacetonic extract of root barks from *Annona senegalensis* Pers. on biochemical parameters of rats are presented in Figure 4. The extract produced nonsignificant effects on plasma levels of liver enzymes (ALT, AST, and ALP), total protein, and albumin compared to the control group. No statistical difference (*P* > 0.05) was found compared to the control group.

**Figure 5:**
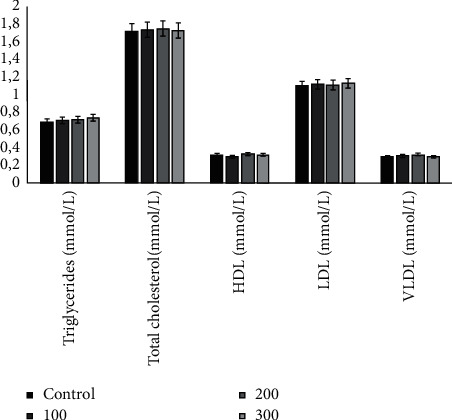
Effects of hydroacetonic extract of root barks from *Annona senegalensis* Pers. on the lipid profile of rats. HDL, high-density lipoprotein; LDL, low-density lipoprotein; and VLDL, very-low-density lipoprotein. No statistical difference (*P* *>* 0.05) was found compared with the control group.

*(2) Effects on Lipid Profile*.  Figure 5 shows that the lipid profile was not significantly altered in rats treated with hydroacetonic extract compared to the control group. No statistical difference (*P* > 0.05) was found compared to the control group.

**Figure 6:**
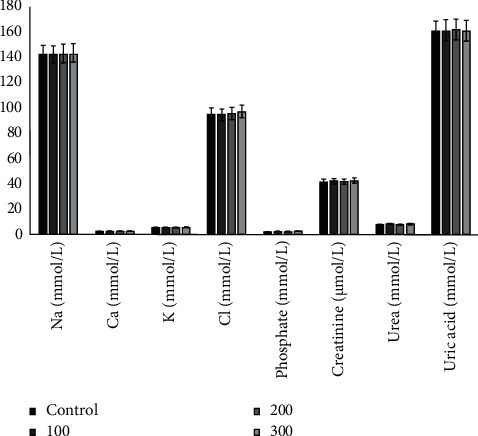
Effects of hydroacetonic extract of root barks from *Annona senegalensis* Pers. on rat kidney indices. No statistical difference (*P* *>* 0.05) was found compared with the control group.

*(3) Effects on Renal Function Parameters*. Analysis of renal function parameters in rats treated with the extract showed a nonsignificant reduction in concentration at all doses used compared to the control group. There was no statistical difference (*P* > 0.05) compared to the control group (Figure 6).

### 3.4. In Vitro Anti-Inflammatory Activity

**Figure 7:**
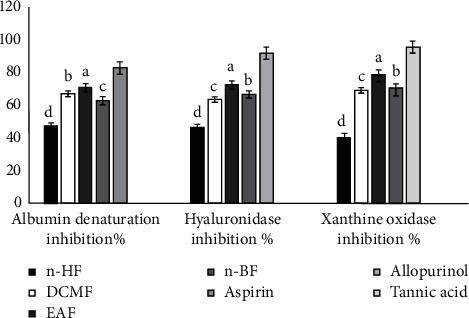
In vitro anti-inflammatory activity of fraction extracts of root barks from *Annona senegalensis* Pers. Values are mean ± SD (*n* = 3). Within each variable, means with different superscripts are statistically different (*P* < 0.05).

Anti-inflammatory activity was assessed by the percentages of hyaluronidase inhibitory activity, protein denaturation inhibition, and xanthine oxidase inhibition, and the results are described in Figure 7. The inhibition of albumin denaturation ranged from 46.8 ± 1.60% to 70.6 ± 1.54, the inhibition of hyaluronidase from 46.12 ± 2.23% to 72.18 ± 2.00%, and the inhibition of xanthine oxidase ranged from 40.36 ± 1.61% to 78.67 ± 1.54. According to these results, the strongest inhibition was obtained by the ethyl acetate fraction followed by the dichloromethane fraction compared to the control compounds. The ethyl acetate fraction also showed the highest percentage inhibition among the extracted fractions (Figure 7).

### 3.5. In Vivo Antiulcerogenic Effect of the Ethyl Acetate Fraction

#### 3.5.1. Antiulcer Activity in Pyloric Ligation-Induced Ulcers

**Figure 8:**
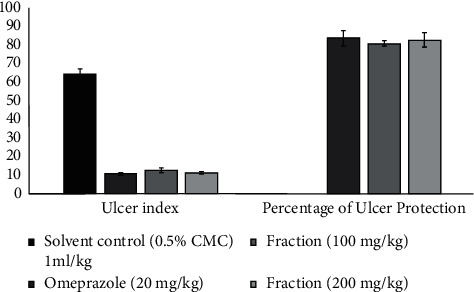
Effect of ethyl acetate fraction on the pylorus ligated rat model indicating ulcer index and percentage ulcer protection. Values are mean ± SEM (*n* = 6); ^*∗*^*P* value <0.05; ^*∗∗*^*P* value <0.01; and ^*∗∗∗*^*P* value <0.001.

The results of the effect of the ethyl acetate fraction of *Annona senegalensis* in the pyloric ligation model are presented in Figure 8. The results indicate that the fraction at dose levels of 100 mg/kg and 200 mg/kg produced a significant decrease in the ulcer index, which was also evidenced by a significant increase in the percentage of ulcer protection at the dose levels 80.94% and 82.81%, respectively. The activity was comparable and equipotent to that of the standard drug omeprazole (83.82%).

#### 3.5.2. Gastric Fluid Study

**Figure 9:**
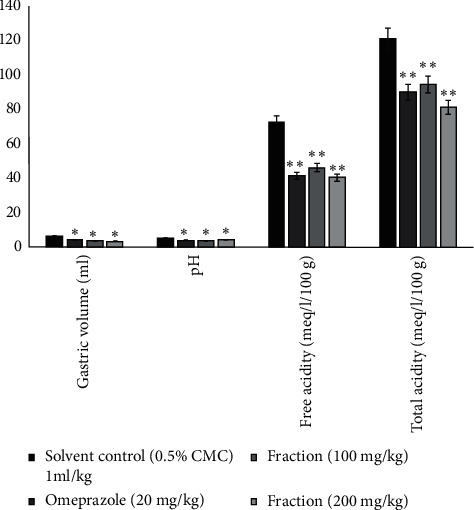
Effect of ethyl acetate fraction on the pylorus ligated rat model indicating pH, gastric volume, free acidity, and total acidity of gastric juice. Values are mean ± SEM (*n* = 6); ^*∗*^*P* value <0.05 and ^*∗∗*^*P* value <0.01 compared with the corresponding control.

Biochemical parameters such as secretions, gastric volume, pH, free, and total acidity were evaluated.  Figure 9 shows the results of the determination of the gastric volume of the ethyl acetate fraction on the treated groups. It shows that there was a significant decrease in gastric juice volume. The activity was comparable and equipotent with the effects of omeprazole (*P* < 0.01). The results of the determination of the gastric pH of the ethyl acetate fraction (Figure 9) in the treated groups indicated that there was a significant increase in the pH of the gastric juice. The activity was comparable and equipotent with the effect of omeprazole (*P* < 0.01). The results of the estimation of the free and total acidity of gastric juice of the ethyl acetate fraction (Figure 9) in the treated groups indicated that there was a significant decrease in the free and total acidity of gastric juice compared to the control animals.

#### 3.5.3. Ethanol-Induced Gastric Ulcer Model

**Figure 10:**
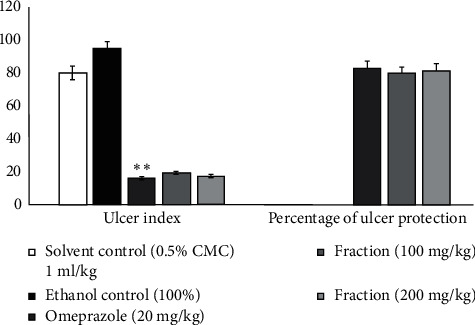
Effect of ethyl acetate fraction (EAF) from *Annona senegalensis* Pers. on the ethanol-induced ulcer model indicating ulcer index and percentage ulcer protection. Values are mean ± SEM (*n* = 6); ^*∗*^*P* value <0.05; ^*∗∗*^*P* value <0.01; and ^*∗∗∗*^*P* value <0.001.

The effect of the ethyl acetate fraction (EAF) of *Annona senegalensis* Pers. on the ethanol-induced ulceration model is shown in Figure 10. The results show that the extracts of the tested fraction have a protective activity for the gastric mucosa, at the doses of 100 mg/kg and 200 mg/kg of fractions. The results of the ethanol-induced ulceration model suggest that the ethyl acetate fraction (EAF) at the dose levels of 100 mg/kg and 200 mg/kg produced a significant decrease in the ulceration index (*P* < 0.001), which was also evidenced by no statistical difference (*P* > 0.05) in the percentage of protection against ulcers at the dose levels of 100 mg/kg and 200 mg/kg (79.66% and 81.52%), respectively. The activity at both dose levels was similar and equipotent to that of omeprazole (82.92%), which is not statistically different from the treatment group (*P* > 0.05).

## 4. Discussion

Nowadays, it is clear that traditional medicine occupies a major role in health habits. The use of *Annona senegalensis* Pers. in primary healthcare can no longer be denied from an ethnobotanical point of view. On the other hand, few studies have been conducted on the biological activities of this plant. The experimental extract of *Annona senegalensis* Pers. root bark has been shown to be nontoxic. It was observed that no signs of toxicity were observed in rats exposed to different doses of root bark extract up to 3400 mg/kg body weight. Indeed, according to some studies, substances with an LD_50_ between 5 mg/kg bw and 5000 mg/kg bw were found to be slightly toxic [29].

With regard to subacute or long-term toxicity, we considered counting with the body weights and vital organs of the animals during 28 days of the study. Indeed, several toxicological studies have shown that vital organ weights are a sensitive indicator of xenobiotic effects [30]. The results showed that, during 28 days of the experiment, the food consumption and body weight of the animals in the test groups did not show any significant change compared to the control group. Furthermore, the vital organ weights of the animals in the test groups were almost identical to those of the control groups, with small variations in some groups that could be considered insignificant and of no consequence. A recent study has shown that the relative weight of organs is an important indicator of the physiological and pathological state of the animals. It is accepted that the hydroacetonic extract of *Annona senegalensis* was not toxic to the vital organs of experimental animals. Regarding the toxicity of the extract on haematological parameters, it can be assumed that *Annona senegalensis* extract did not show significant changes in haematological parameters in the experimental groups, compared to the control group. Hematopoiesis can be negatively affected by synthetic molecules or phytomedicines. Haematological parameters are reliable indicators of the physiological and pathological state of living organisms [31]. The absence of alterations in haematopoietic parameters may indicate that hydroacetonic extract does not adversely affect the bone marrow, since all blood cells originate from haematopoietic stem cells in the bone marrow [32].

It is known that the liver and kidney function parameters are the site of functional, purification-based metabolism with significant elimination in living beings. Therefore, the analysis of their functions is extremely important for the toxicological study of xenobiotics [33]. The results of the current study showed that the hydroacetone extract of *Annona senegalensis* did not negatively affect liver and kidney parameters. Similarly, the analysis of the results presented suggests that there was no variation in the lipid profile of the animals in the treated groups compared to the control rats. These results may indicate that the hydroacetone extract did not have a negative effect on the phenomena that can lead to an increase in plasma lipoprotein levels, namely, low-density lipoproteins (LDLs), which are responsible for the biosynthesis of hepatic cholesterol, which may affect the catabolic mechanism of LDL cholesterol in the blood and liver [34].

The presented results of the anti-inflammatory potential of *Annona senegalensis* root barks showed numerous anti-inflammatory properties of the ethyl acetate fraction in vitro, namely, inhibition of protein denaturation, inhibition of hyaluronidase, and inhibition of xanthine oxidase. Protein denaturation is a process by which proteins lose certain structures, such as tertiary and secondary structure [35]. The results of the presented research work indicate that the experimental fraction extract significantly inhibited protein/albumin denaturation. The hyaluronidase inhibition test of the fractionated extracts showed moderate antihyaluronidase activities compared to tannic acid (reference substance). The proinflammatory enzyme xanthine oxidase also plays a major role in the metabolic disease called gout, which is closely associated with inflammation and other inflammatory-mediated diseases due to the formation of free radicals during the enzyme's catalytic function. Therefore, inhibition of this enzyme is considered a target for the management of diseases associated with oxidative stress and inflammation [36]. In the current study, extracts from the fractions studied showed significant xanthine oxidase inhibitory activity (*P* < 0.05). The ethyl acetate fraction showed the highest xanthine oxidase inhibitory activity compared to the other fractionated extracts. The results are better than those obtained by Perera et al. [37]. The anti-inflammatory activity of the experimental fraction extract could be explained by the presence of some specific biologically active compounds. Aqueous and ethanolic extracts of *Annona senegalensis* leaves and roots were found positive for flavonoids, tannins, cardiac glycosides, saponins, alkaloids, steroids, and volatile oils, but negative for saponin glycosides and anthraquinones [38]. Phenolic compounds, including flavonoids, have anti-inflammatory properties [16].

Herbal medicines are good candidates for the treatment and prophylaxis of gastroprotections worldwide [39]. Herbs can be used to reduce gastric acid secretion or to improve mucosal defense mechanisms by increasing mucus production [40]. In this case, the ethyl acetate fraction obtained from *Annona senegalensis* showed a high gastroprotective potential compared to synthetic omeprazole. Therefore, the reduction of the acidity of gastric juice by the ethyl acetate fraction may also be due to its antihistaminic effect. Indeed, the aqueous extract of *Annona senegalensis* root has been studied for its inhibitory activity towards *Bitis arietans* venom protease and phospholipase *A*_2_ (PLA_2_) activity. The histamine potential of *Annona senegalensis* root extract was confirmed [41]. The antihistaminic and gastroprotective effect of the ethyl acetate fraction obtained from the crude methanolic extract of *Muntingia calabura* leaves were also observed [42].

It is well known that gastric acid is a significant factor in the genesis of ulceration in pyloric ligated rats. The presented study clearly shows that the ethyl acetate fraction (EAF) of *Annona senegalensis* decreases gastric acid in a dose-dependent manner. These results are similar to those obtained by Sonali et al. [43]. Thus, among the samples tested, the best result was obtained with the ethyl acetate fraction at an optimal dose of 200 mg/kg which was potentially effective compared to the standard drug, omeprazole.

## 5. Conclusions and Recommendations

The results obtained in this study indicate that the oral administration of the hydroacetone extract is relatively safe and has low toxicity. The ethyl acetate fraction of *Annona senegalensis* could be identified as a potential candidate for the development of a new anti-inflammatory herbal drug, which deserves further study. However, in this study, the bioactive fraction of ethyl acetate showed protection against the characteristic lesions produced by ethanol administration. The antiulcer potential of the bioactive fraction may be due to both reduction of gastric acid secretion and gastric cytoprotection. Further studies are needed for their fraction to clarify the mechanism of action on gastric acid secretion and gastric cytoprotection. However, the presented study confirmed that treatment with the bioactive fraction reduced ethanol-induced ulcer in a dose-dependent manner and that, at the highest dose (200 mg/kg), the effect was similar to that of the reference drug. In conclusion, the ethyl acetate fraction was found to have anti-inflammatory and antiulcerogenic properties. The ethyl acetate fraction at the dose of 200 mg/kg showed the highest level of cytoprotection. The depletion of inflammation may have occurred due to the high content of flavonoids, triterpenoids, steroids, saponins, and tannins. However, the mechanisms behind these events are still unclear. Therefore, further experiments should be undertaken to identify which phytoconstituents and mechanisms are involved in the actions illustrated by the results.

## Data Availability

The datasets generated and/or analysed during the study are available from the corresponding author on reasonable request.
